# Spirit of the British and American Periodicals

**Published:** 1839

**Authors:** 


### Spirit of the British and American Periodicals.

---

### Severe Symptoms from the Bite of a Spider on the Glans Penis.

This case is related in the American Journal of the Medical Sciences,* by Dr. Hulse, of the United States Navy.

*Case.*—Mr. Q. of Pensacola, of short stature, dark complexion, and muscular frame, on the 7th of August, 1838, "while in the privy, perceived himself to be stung by a spider on the glans penis. The pain, which was not great at the moment, continued to increase till 1 P.M. an hour after the accident, when it had become extreme, and I was called to see the patient. I found him lying upon a cot, and writhing under the most acute suffering. The place where the sting was made, showed no marks of irritation nor swelling. I however applied to it a strong solution of carbonat. potass, which I happened to have about me, and ran to the apothecary's for medicine. My absence lasted but a few minutes, and on my return, I found him vomiting with great violence, and complaining of deep-seated pain in the abdomen, extending up into the chest, and of sensations of choking and suffocation. The vessels of the neck and face were greatly distended, and of a dark hue. I opened a vein in the arm and let blood copiously through a large orifice, and commenced immediately to give aqua ammoniæ and laudanum in doses of a teaspoonful of each every ten minutes, which were ejected as often from the stomach—pains and spasms along the spine and extremities now came on, and the agony and anxiety were, if possible, increased. Strong volatile liniment, tinct. cantharides, and spirits terebinth. were alternately applied to every part of the body by the patient's numerous friends who had assembled round him, and common injections were administered as frequently as they conveniently could be, with a view to open the bowels. The ammonia and laudanum were assiduously plied and occasionally some tinct. camphoræ, likewise; at the suggestion of Dr. Edwards of the navy who was called in, the oleum olivarum was freely administered. At 3 P.M. the paroxysms of pain came on at longer intervals, and the vomiting was less urgent, but the intensity of the pain when present, was undiminished. The principal medicine relied on, viz. the ammoniæ and laudanum, were continued every half hour, and at about five o'clock, after the exhibition of fifteen injections, fæcal evacuations were obtained from the bowels. The patient became much easier in the course of the evening, and was able to retain a dose of castor oil, which purged him freely; but the pain in the legs continued through the night, which he passed without sleep.

On the subsequent day, sinapisms were applied to the legs without effect, and the evening brought little or no mitigation of the pain. Veins were now opened in both feet, which were placed in warm water, and the blood was allowed to flow till an impression was made on the pulse. In an hour after the bleeding, the patient enjoyed perfect ease; he slept well that night, and on the following day was able to walk about the house. He recovered in health very speedily."

"I saw," adds Dr. Hulse, "several spiders in the place where he received the sting. They were of large size, of a dark brown colour, covered with hairs over the legs and body."

---

* May, 1839.

In this case four ounces of laudanum and an equal quantity of aqua ammoniæ were administered in the space of four hours.

